# Supplementary material for: Rainfall and Temperature Influences on Childhood Diarrhea and the Effect Modification Role of Water and Sanitation Conditions: A Systematic Review and Meta-Analysis
Source: Int J Environ Res Public Health. 2024 Jun 24;21(7):823. doi: 10.3390/ijerph21070823 (PMC11276699; doi:10.3390/ijerph21070823)
Supplement: Supplementary file 1 [file ijerph-21-00823-s001.zip › ijerph-2952501 - Supplementary Material ( Figures).docx-revised.pdf]

Supplementary Figures

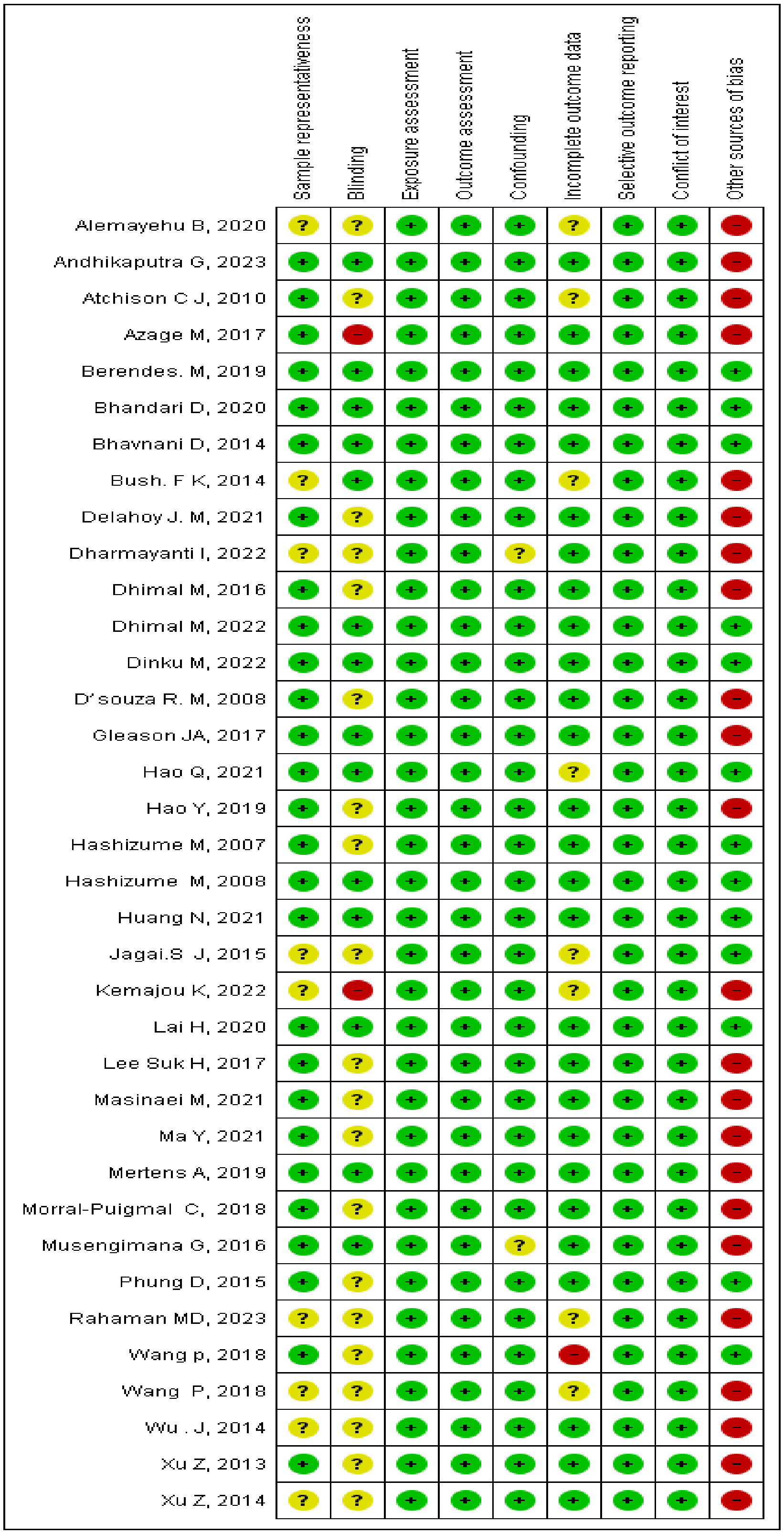

Figure S1: Risk of bias summary: review authors' judgments about each risk of bias item (using a green symbol for low risk of bias, yellow for unclear, and red for high risk of bias) for each included study [23–58].

Supplementary Figures

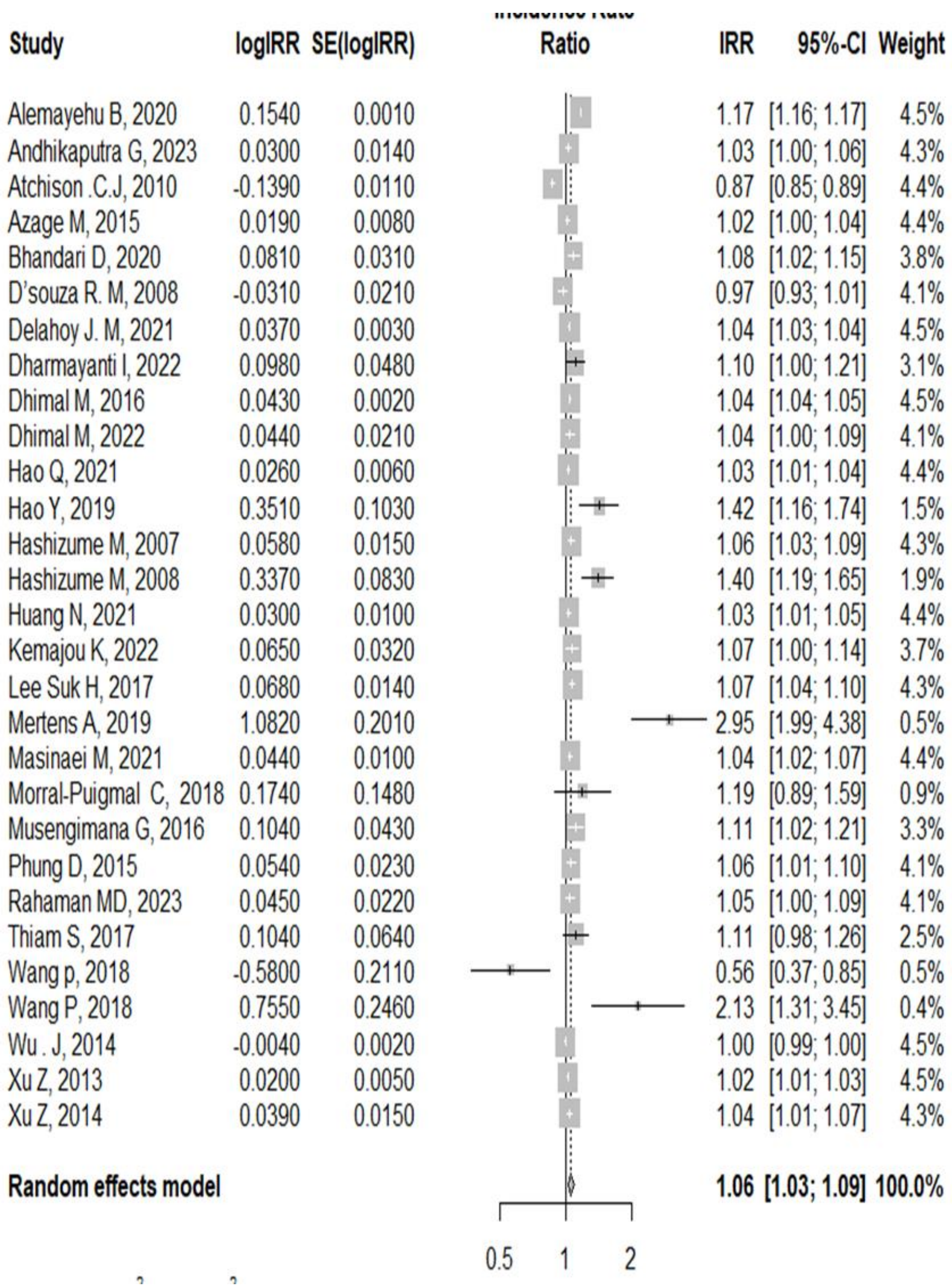

Figure S2: Forest plot of the systematic review of the association between temperature and childhood diarrhea [23–27, 31–35, 37–41, 43, 45, 47, 49, 50–58].

Supplementary Figures

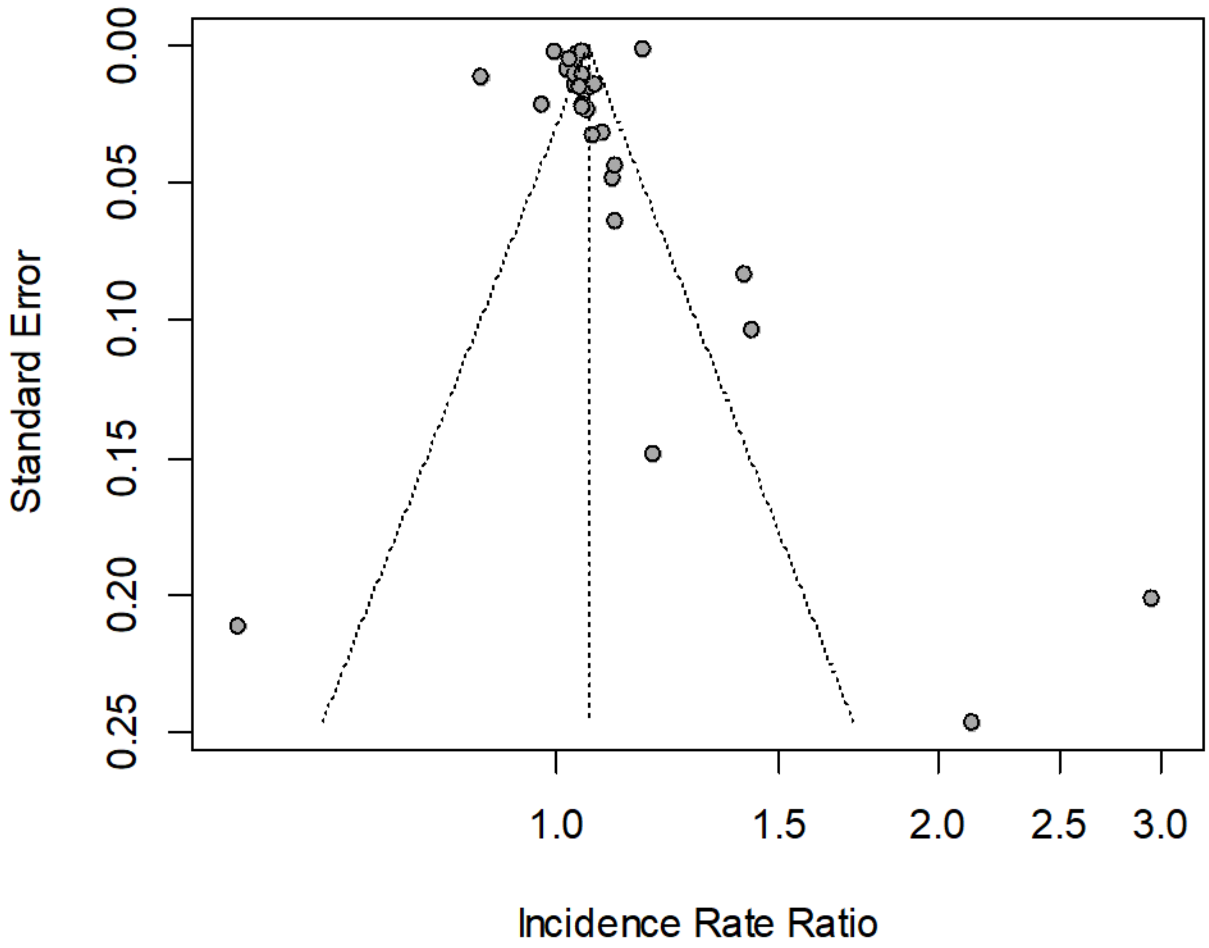

Figure S3: Funnel plot of the systematic review of the association between temperature and childhood diarrhea [23–27, 31–35, 37–41, 43, 45, 47, 49, 50–58].

Supplementary Figures

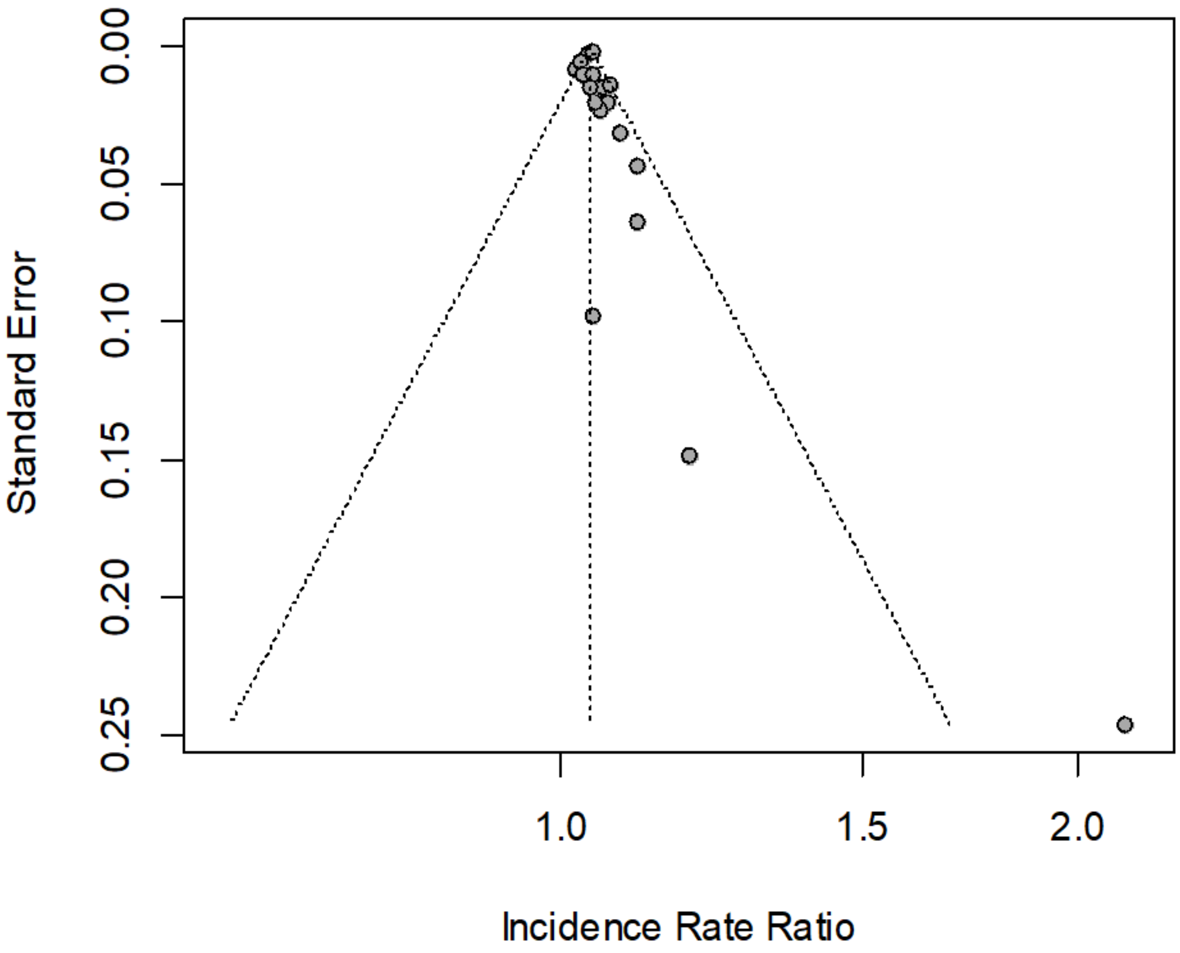

Figure S4: Funnel plot of the systematic review of the association between temperature and childhood diarrhea after removing those articles that contributed high heterogeneity [24, 26–27, 32–33, 35, 37, 39, 41, 43, 45, 47, 50–55, 58].

.

Supplementary Figures

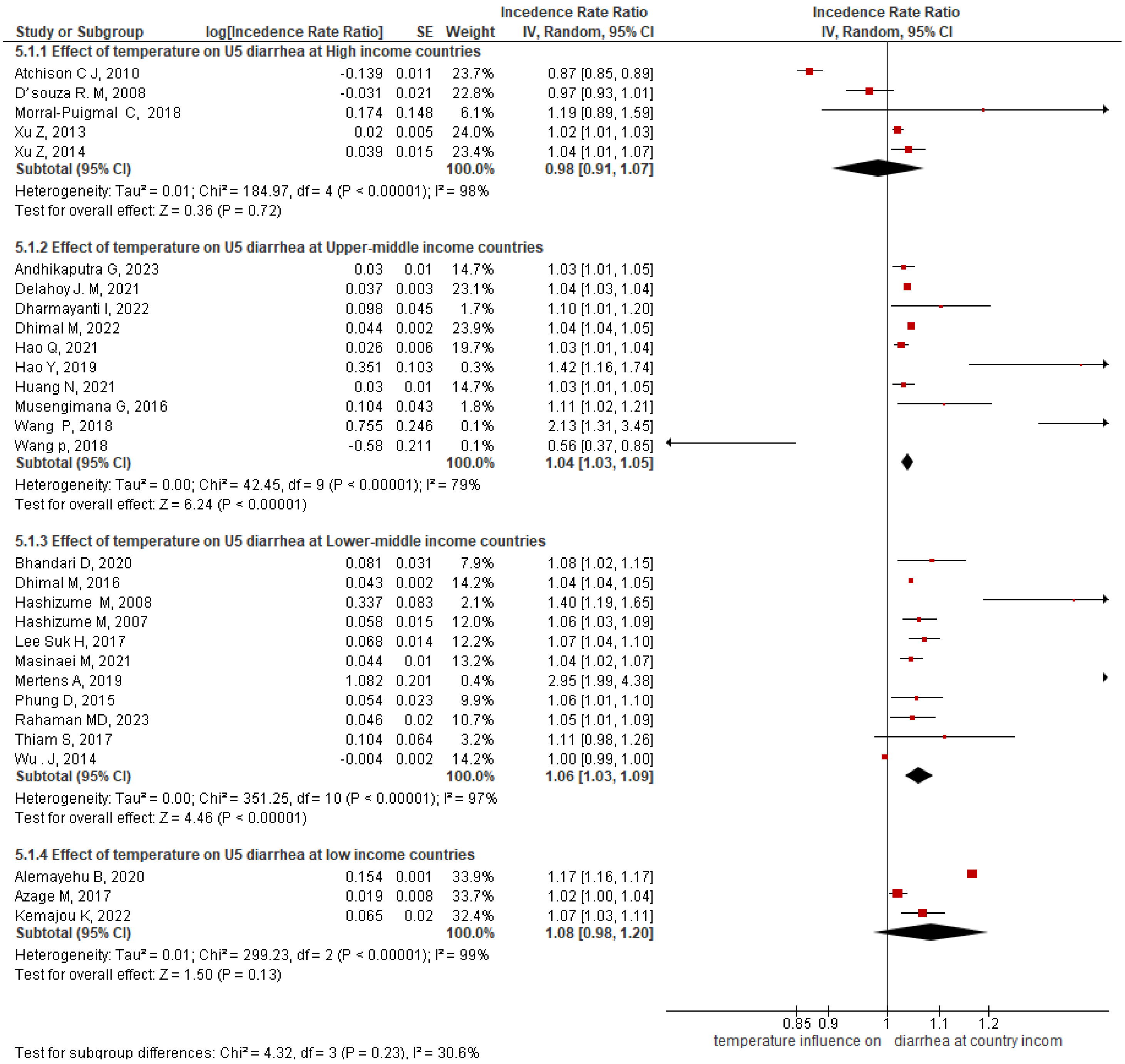

Figure S5: A forest plot of the systematic review of the association between temperature and childhood diarrhea at country income level [23–27, 31–34, 36–41, 43, 45, 47, 49, 50–58].

Supplementary Figures

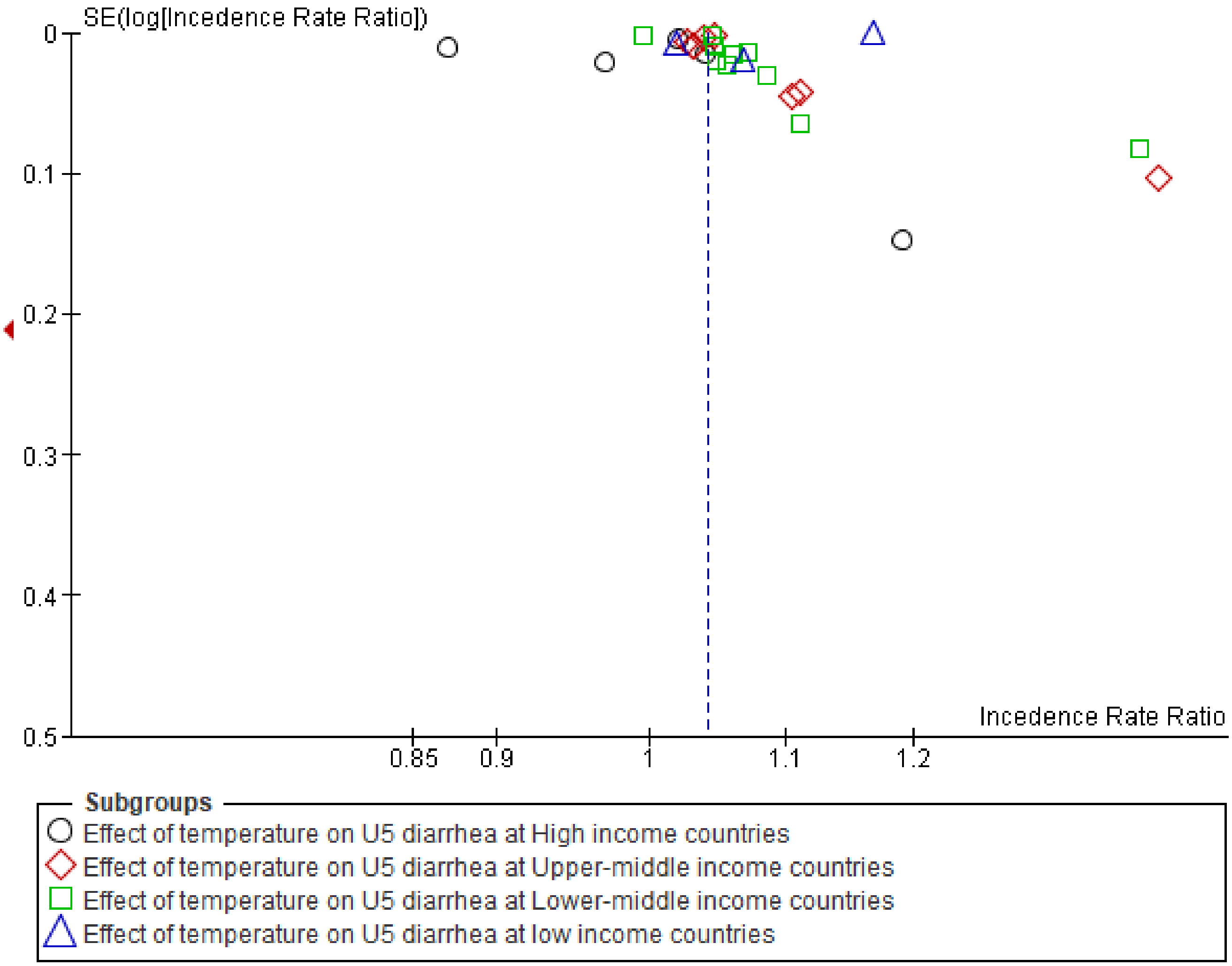

Figure S6: Funnel plot of the systematic review of the association between temperature and childhood diarrhea at country income level [23–27, 31–34, 36–41, 43, 45, 47, 49, 50–58].

Supplementary Figures

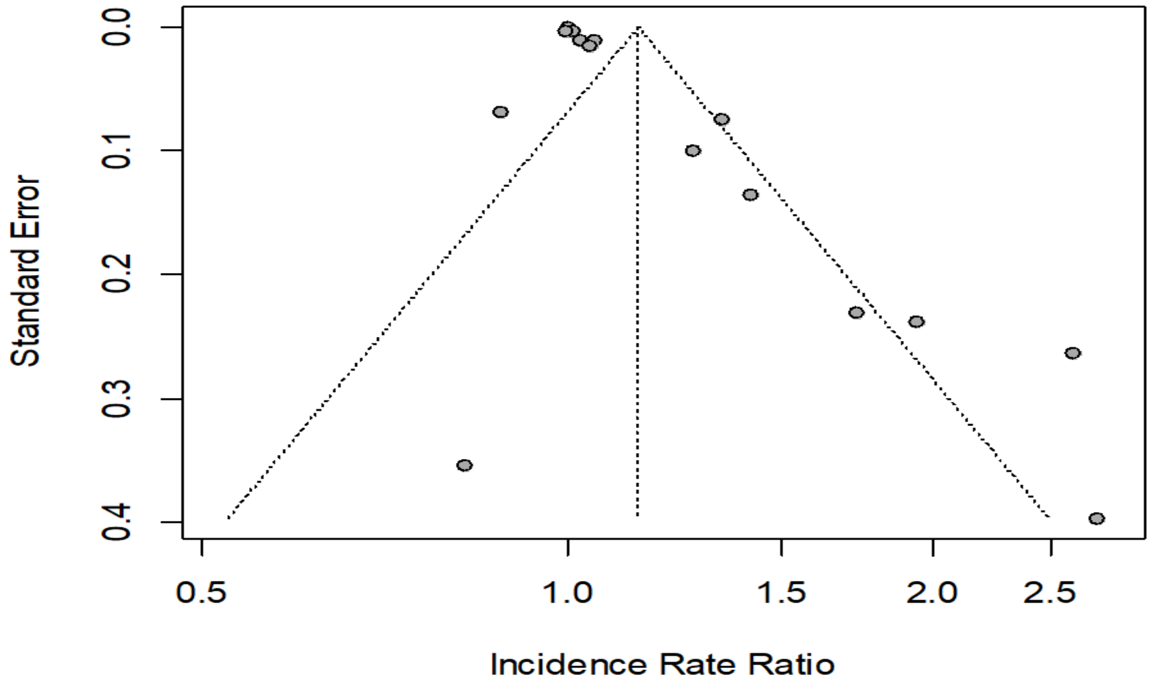

Figure S7: Funnel plot of the systematic review of the association between rainfall and childhood diarrhea [23, 26–29, 34, 36, 39, 44–46, 49, 54–55, 58].

.

Supplementary Figures

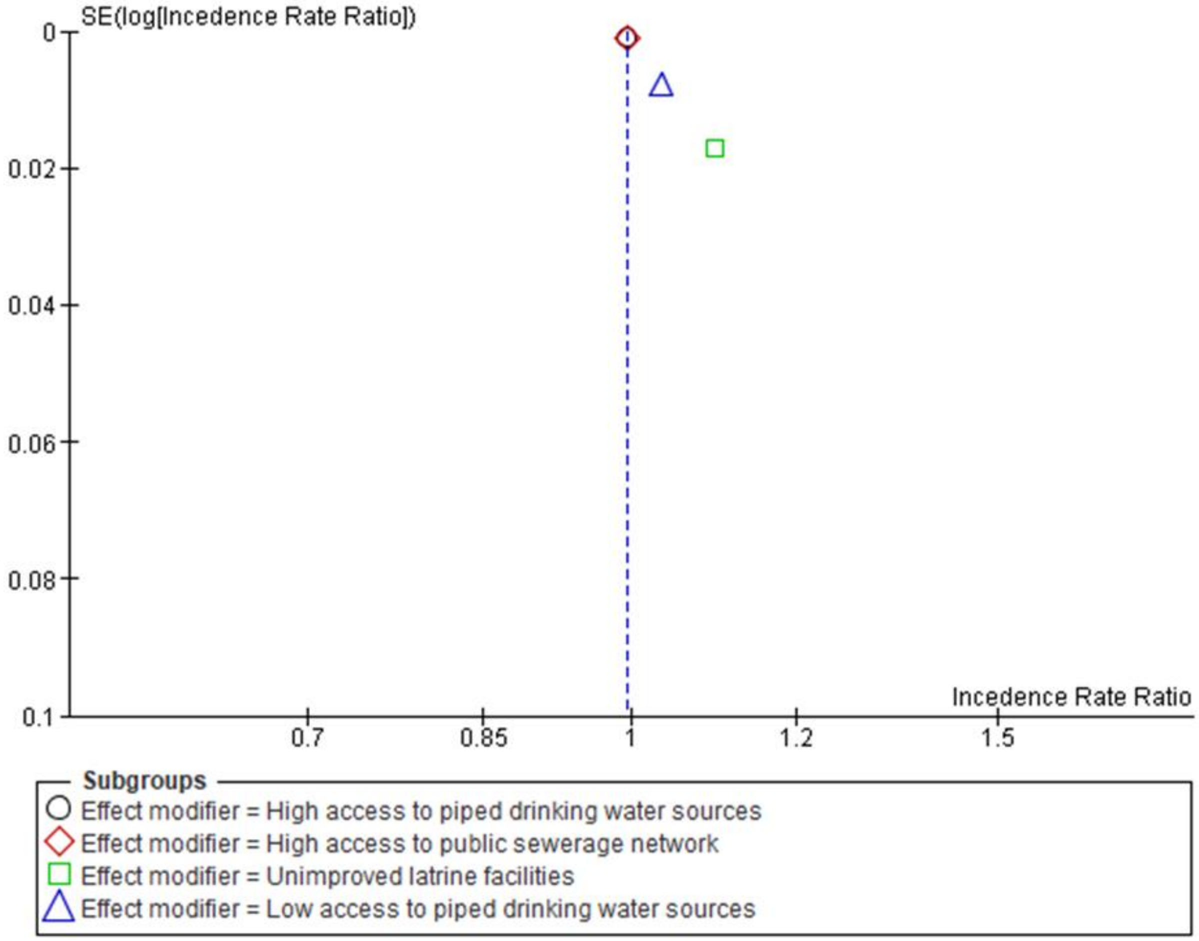

Figure S8: Funnel plot of meta-analysis on the modified association of temperature with childhood diarrhea due to water and sanitation conditions [47, 39, 32].

Supplementary Figures

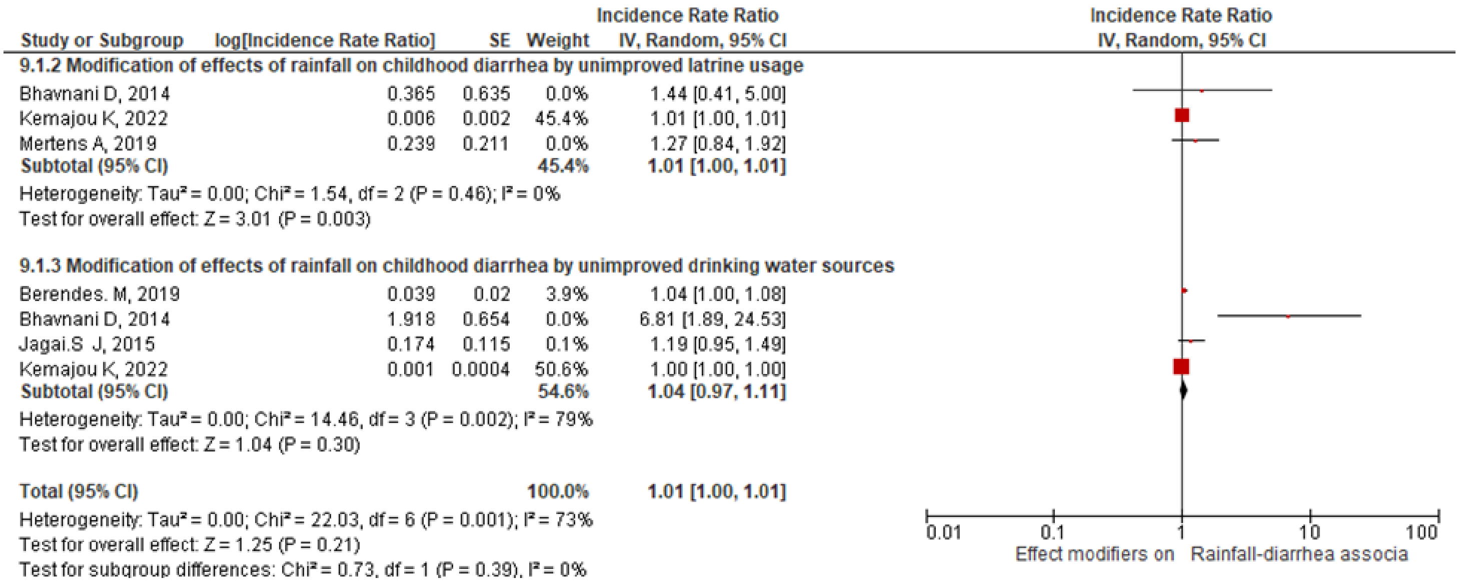

Figure S9: Forest plot of meta-analysis on the modified association of rainfall with childhood diarrhea due to water and sanitation conditions (without removing any study) [28, 30, 42–43, 49].

Supplementary Figures

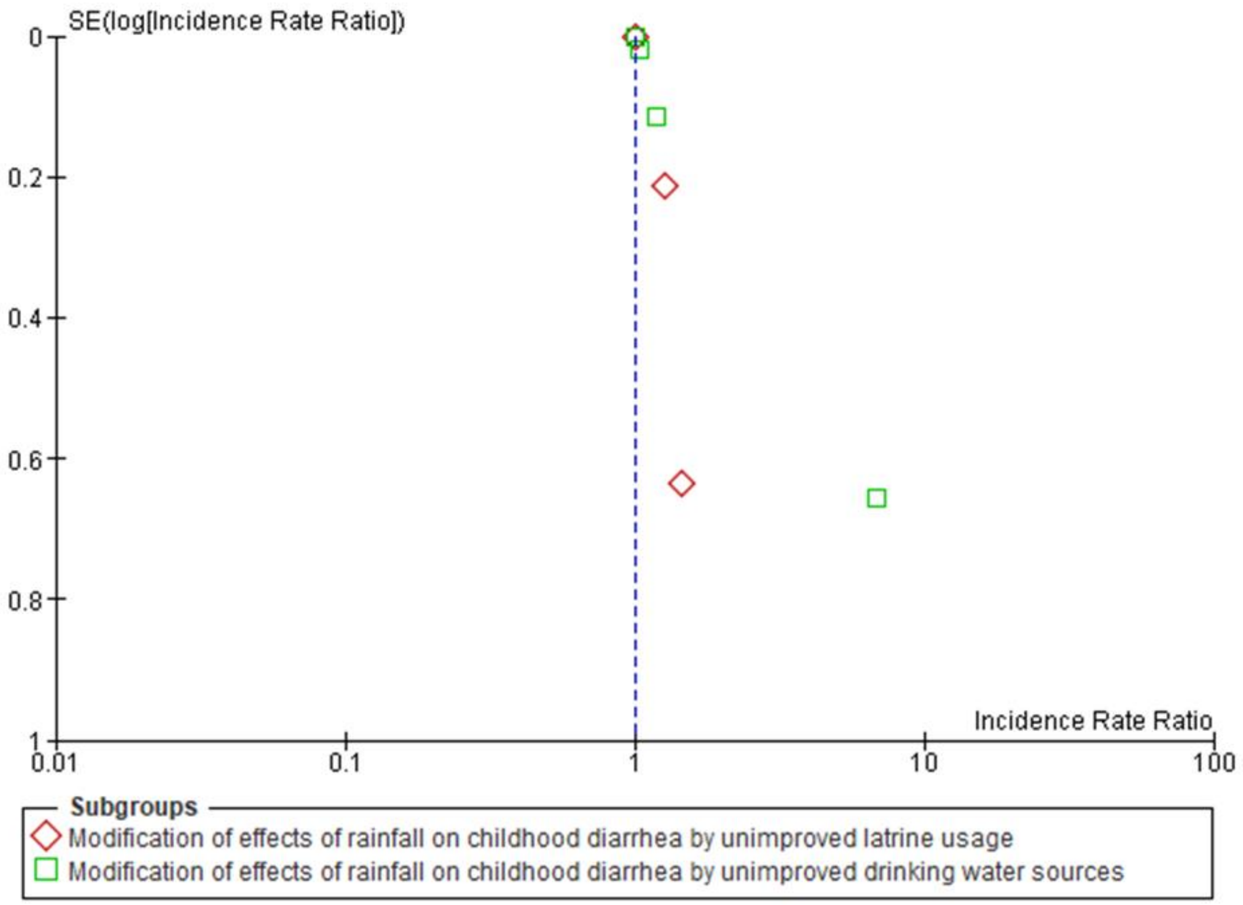

Figure S10: Funnel plot of meta-analysis on the modified association of rainfall with childhood diarrhea due to water and sanitation conditions (without removing any study) [30, 28, 42–43, 49].

Supplementary Figures

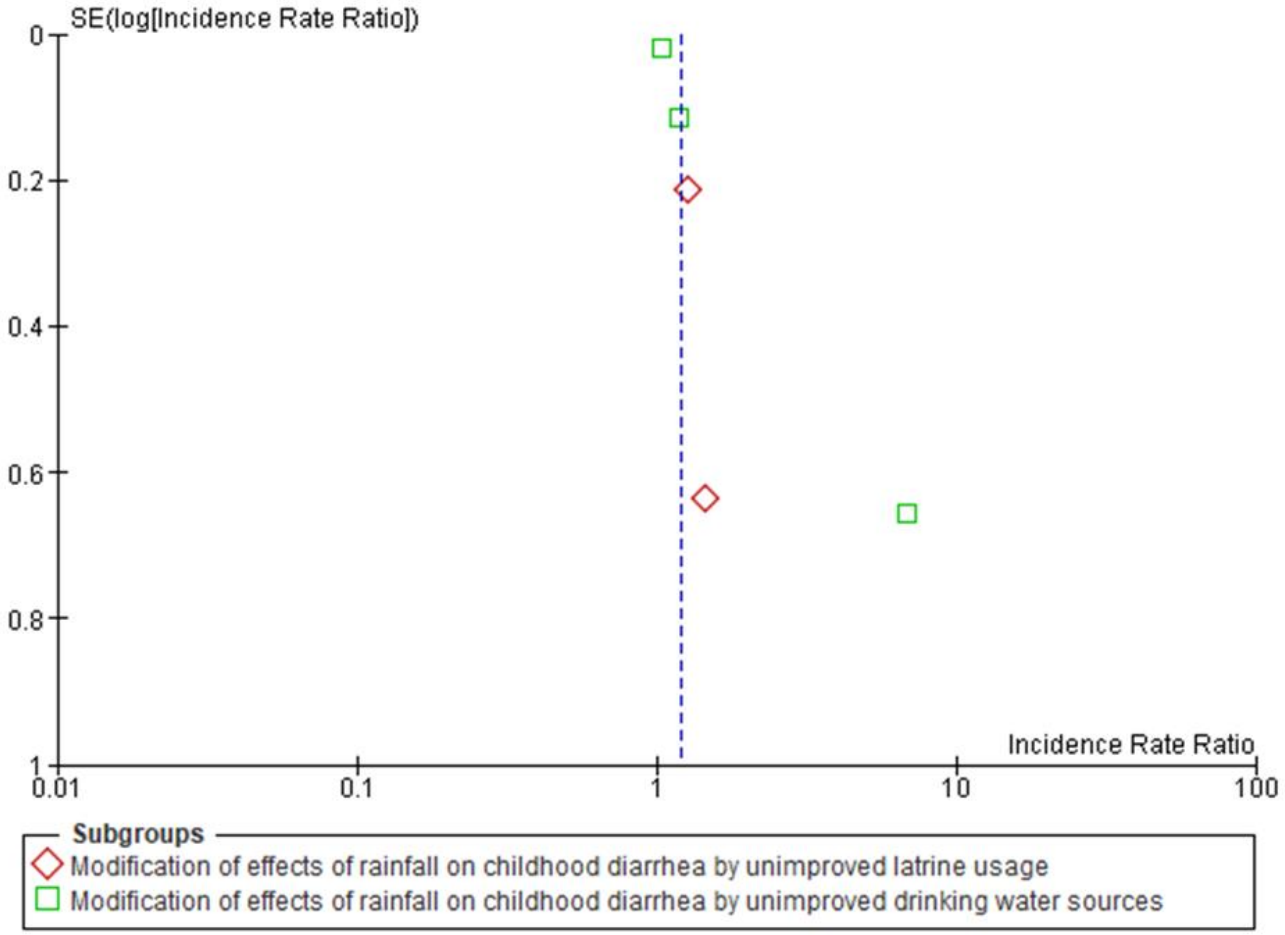

Figure S11: Funnel plot of meta-analysis on the modified association of rainfall with childhood diarrhea due to water and sanitation conditions [28, 49, 30, 42].
